# Coinfection of avian hepatitis E virus and different serotypes of fowl adenovirus in chicken flocks in Shaanxi, China

**DOI:** 10.1128/spectrum.01338-24

**Published:** 2024-12-17

**Authors:** Yuan Zhang, Huanyu Xu, Yinuo Tian, Jirong Tang, Huanqing Lin, Yani Sun, Qin Zhao, En-Min Zhou, Yiyang Chen, Baoyuan Liu

**Affiliations:** 1 Department of Preventive Veterinary Medicine, College of Veterinary Medicine, Northwest A&F University, Yangling, Shaanxi, China; 2 Kongtong Animal Disease Prevention and Control Center, Pingliang, Gansu, China; Taichung Veterans General Hospital, Taichung, Taiwan

**Keywords:** hepatitis E virus, fowl adenovirus, coinfection, epidemiological investigation

## Abstract

In poultry, fowl adenovirus (FAdV) and co-infected viruses (such as avian hepatitis E virus, aHEV) are likely to cause decreased egg production, inclusion body hepatitis, and pericardial effusion syndrome. From July to September 2023, eight poultry farms of commercial broilers and commercial layers suffered from increased mortality, decreased egg production, and the presence of hydropericardium-hepatitis syndrome-like gross lesions in Shaanxi province, China. To determine the source of the infection, the viruses of aHEV, FAdV, avian leukosis virus (ALV), Marek’s disease virus (MDV), Newcastle disease virus (NDV), and H9N2 avian influenza virus (AIV) were detected. A total of 240 liver samples were collected from eight farms and detected by molecular etiology methods. The samples were tested positive for FAdV and aHEV and negative for NDV, MDV, ALV, and H9N2 AIV. Phylogenetic analysis based on the hexon loop-1 gene showed that these isolates were clustered into three distinct serotypes: FAdV serotype 4 (FAdV-4), FAdV-8a, and FAdV-8b. The partial ORF2 gene sequences obtained in this study shared the highest identity (76%–97%) with corresponding sequences of other known avian HEV isolates and belonged to gene type 3 avian HEV. Meanwhile, the isolated FAdV in this study could multiply in leghorn male hepatoma (LMH) cells. Overall, the disease may be mainly caused by FAdV and aHEV coinfection on these farms, and this is the first study to discover FAdV-4/8a/8b and avian HEV coinfection in a farm in China.

**IMPORTANCE:**

Last year, eight poultry farms of commercial broilers and commercial layers suffered a serious disease in Shaanxi province, China. After molecular etiological investigation, the disease may be mainly caused by fowl adenovirus (FAdV) (FAdV-4, FAdV-8a, and FAdV-8b) and avian hepatitis E virus (aHEV) (genotype 3 avian HEV) coinfection on these flocks, and this is the first study to find FAdV-4/8a/8b and avian HEV coinfection in a farm. It provides a valuable foundation for the prevention and control of FAdV and avian HEV coinfection in chicken farms.

## INTRODUCTION

Fowl adenoviruses (FAdVs) are non-enveloped, double-stranded DNA linear viruses belonging to the genus Adenoviridae and the family Aviadenovirus (1). According to molecular structure and serum cross-neutralization test, FAdV is classified into five species (FAdV-A to FAdV-E) and 12 serotypes (FAdV-1 to 8a and 8b to 11) (2). FAdV infection is usually related to inclusion body hepatitis (IBH), hepatitis-hydropericardium syndrome (HHS), and gizzard erosion (GE) which are generally caused by specific serotypes (3–5). IBH is characterized by focal or diffuse necrosis in the liver tissue with characteristic hemorrhages and macroscopic lesions and is associated mainly with FAdV serotypes 2, 11, 8a, and 8b (6). FAdV-4 is the primary causative agent of HHS, which was first reported in Angara Goth, Pakistan in 1987 and subsequently outbroke in many other countries (5, 7). GE is characterized by multifocal ulcerations and hemorrhages in the mucosa gizzard of chickens and is mainly associated with FAdV-1 infection (8, 9). Although the major causative agent of GE is FAdV-1, a few cases of FAdV-4, FAdV-8 (FAdV-8a and 8b), and FAdV-11 strains have been implicated (10, 11). Moreover, FAdV can cause immunosuppression in infected birds and generally co-infect with other viruses, which would cause more serious diseases and higher mortality (12). Outbreaks of IBH and HHS were sporadically reported in China, which mainly related to infection of FAdV-4, 8b, and 11. However, since 2015, severe HHS induced by a novel hyper-virulent FAdV-4 has been widespread in commercial chicken farms across China, with a high mortality rate of 30%–90%, causing huge economic loss to the poultry industry (13).

As the causative agent of big liver and spleen disease and hepatitis-splenomegaly syndrome (HSS) in chickens, avian hepatitis E virus (aHEV) is characterized by increased mortality (1%–4%), decreased egg production (10%–40%), enlarged livers and spleens in broiler breeders and laying hens 30–72 weeks of age, which leads to significant economic losses in the poultry industry (14–16). However, accumulating evidence suggests that this virus may be an opportunistic virus. Recently, epidemiological investigation has shown that coinfection, particularly dual infection, is common in farms with HSS (17). When two or more viruses, particularly immunosuppressive pathogens such as avian leukosis virus (ALV), Marek’s disease virus (MDV), reticuloendotheliosis (REV), FAdV, and chicken infectious anemia virus, simultaneously or successively infect chickens (12), the pathogens will produce synergy, resulting in more serious clinical signs than mono-infection (17–19).

From July to September 2023, eight poultry farms of commercial broilers and layers emerged successively, with mortality increasing and egg production decreasing, especially in 30–100-day-old layers with 20%–65% mortality. The peak of death occurred 3–7 days after the onset of the disease, and the course could last 5–20 days. Additionally, the chickens showed flabby hearts with straw-colored fluid in the pericardial sac and a swollen liver with foci of hemorrhage and/or necrosis. To confirm the causative agent of disease in these chicken flocks, 240 liver samples were collected and analyzed using molecular etiology methods. The results revealed that avian HEV and FAdV were coinfections in these flocks, and interestingly, FAdV-4/8a/8b and avian HEV were infected on a farm.

## MATERIALS AND METHODS

### Clinical observation and sample collection

The chickens in these farms exhibited reduced weight gain, stunted growth, and listlessness associated with loose, yellow-green-colored stools. The peak of egg production was significantly decreased by approximately 20%. No respiratory signs (discharge from the eyes, runny nose, and sneezing/snicking) were observed in these birds. Mortality rates ranged from 10% to 65% in these farms (Table 1), and vaccination details are shown in Table 1. Two hundred forty liver and 240 serum samples were collected from two layer farms and six broiler farms with 30 samples per farm from July to September 2023 in Shaanxi province, China (Table 1).

**TABLE 1:** Detailed history and PCR results of eight farms investigated in Shaanxi province, China in 2023^*a*^

	Farm 1	Farm 2	Farm 3	Farm 4	Farm 5	Farm 6	Farm 7	Farm 8
Farm type	Layer farm	Layer farm	Broiler farm	Layer farm	Layer farm	Broiler farm	Layer farm	Layer farm
Age of birds	38 days	35 days	95 days	310 days	180 days	300 days	80 days	120 days
Vaccination	MDV (D1), V4 + 4/91 + H120 (D1), NDV (D12), IBDV B87 (D21),	MDV (D1),	MDV(D3), NDV LaSota (D15), IBDV (D30), H5N1 (D28, D86)	MDV (D1),	IBDV(D1),	IBV(D1),	IBV(D1),	IBDV(D1),
		NDV^*b*^ LaSota (D12), IBDV B87 (D21), H5N1 (D28),		NDV LaSota (D15), IBDV B87 (D21), H5N1 + H7N9 (D28, D77), AE + FP (D84), NDV (D102)	NDV LaSota (D10), H5N1 (D70), MDV CVI988 (D112)	POX (D15), H5N1 + H7N9 (D21, D154), NDV LaSota(D70), ILT A96 (D124)	NDV LaSota (D7), H5N1 (D14), MDV (D30)	NDV LaSota (D10), H5N1 (D18), NDV + IBV + EDS (D95)
									Mortality	>20%	>65%	>40%	<20%	>25%	<30%	>20%	>10%

	Farm 1	Farm 2	Farm 3	Farm 4	Farm 5	Farm 6	Farm 7	Farm 8
FAdV	+	+	+	+	+	+	+	+
HEV	+	+	+	+	+	+	+	+
MDV	−	−	−	−	−	−	−	−
H9N2 AIV^*b*^	−	−	−	−	−	−	−	−
ALV	−	−	−	−	−	−	−	−
NDV	−	−	−	−	−	−	−	
FAdV serotype detected	4	8a/b and 4	4	4	4	8a	4	4

^*a*^ Mortality: the reported mortality rates are as given by the farmer at the time of sampling. −, negative result, +, positive result.

^*b*^ NDV, Newcastle disease virus; AIV, avian influenza virus.

### Histopathology analysis

Liver samples were fixed in 10% neutral buffered formalin, dehydrated by passing through a graded series of ethanol solutions, embedded in paraffin, sectioned at 4 μm thickness, and stained with hematoxylin and eosin.

### Detection of aHEV antibodies from serum samples

The 240 serum samples were tested with anti-avian HEV IgG antibodies using the indirect ELISA (iELISA) described by Zhao et al. (20). Based on the iELISA assay, the result was considered positive when the optical density values at 450 nm (OD450nm) of testing serum samples were greater than 0.368.

### DNA extraction and PCR assay

Total viral DNA was extracted from all the supernatant liver samples using the AxyPrep Body Fluid Viral DNA Miniprep Kit (Axygen America) according to the manufacturer’s instructions. The ALV, MDV, and FAdV primers were used according to the ALV env gene, MDV meq gene, and FAdV hexon loop-1 gene as previously described, respectively (Table 2) (10, 21, 22).

**TABLE 2:** Primers used in this study

Primers	Sequence (5′–3′)	Size (bp)	Purpose
FAdV	F: CAARTTCAGRCAGACGGT R: TAGTGATGMCGSGACATCAT	896	FAdV hexon gene
HEV	F: TCGCCT(C)GGTAAT(C)ACA(T)AATGC R: GCGTTC(G)CCG(C)ACAGGT(C)CGGCC	242	HEV ORF2 gene
H9N2 AIV	F: TTTTCAGTTCTGCTCGCTCCT R: ATCTTCCGTTGTTATGCTCGT	284	H9N2 AIV HA gene
	F: GAGACCCTAAATAACGAAA R: AGAATACCCAGACCAACT	209	H9N2 AIV NA gene
ALV	F: CGAGAGTGGCTCGCGAGATGG R: ACACTACATTTCCCCCTCCCTAT	1,016	ALV env gene
MDV	F: CGCGAATTCTACAGGTGTAAAGAGATG R: TAACTCGAGTGCTGAGAGTCACAATGC	1,060	MDV meq gene
NDV	F: AGGGACTGAAGAGGAGGATT R: TGAGTGTGATTGTATTAGGTGG	427	NDV F gene

### RNA extraction and RT-PCR assay

Total viral RNA was extracted from the supernatant liver samples using Trizol (TaKaRa, Dalian, China) for the detection of partial avian HEV ORF2 gene, Newcastle disease virus (NDV) F gene, and H9N2 avian influenza virus (AIV) HA and NA gene according to the manufacturer’s instructions. The extracted RNA was then used for cDNA synthesis using TransScript All-in-One First-Strand cDNA Synthesis SuperMix (TransGen Biotech, Beijing, China). Detection of avian HEV, NDV F gene, and H9N2 AIV HA and NA gene was performed according to the procedure previously described (Table 2) (23–25).

### Sequencing and phylogenetic analysis

For the partial hexon gene of FAdV, amplified DNA sequences (896 bp) were compared with each other and with sequences of different serotypes of FAdV strains obtained from GenBank using the MegAlign program of the Lasergene software package (DNASTAR Inc., Madison, WI, USA). Next, a phylogenetic tree was also constructed for FAdV sequences using the MEGA7 software (26). GenBank accession numbers of reference strains of FAdV were KU558760, KU991797, MG547384, MH006602, MK387062, MN781666, HE608152, GU188428, KU342001, AF508950, KT899325, MG856954, MN604721, MN337322, MK875248, MK387061, MF055634, MT759841, AF508957, MK572864, KY968968, MH379428, HQ117904, AF339922, KT862808, AF508947, AF508959, AF508948, and AF508949.

For sequence analysis of the amplified partial ORF2 gene of avian HEV, 242 bp regions were subjected to multiple alignments using the Meg Align program within the Lasergene software package (DNASTAR Inc., Madison, WI). In addition, according to the previous studies, the phylogenetic tree constructed based on the 242 bp is the same as the one based on the complete genomes (27). Therefore, using MEGA7 software, a phylogenetic tree was constructed based on the sequences obtained here and other known avian HEV strains obtained from GenBank. GenBank accession numbers of avian HEV reference strains used for sequence comparisons and phylogenetic tree construction included KP221201, FM872318, AM943646, FM872312, MK050107, AY870825, AY870819, KF511797, JN997392, FM872311, AM943647, FM872313, EU919189, AY535004, EF206691, AY870831, MG737712, KX589065, MG976720, and MN562265.

### Viruses isolation

FAdV isolates, CHN-SX-FAdV-8a, CHN-SX-FAdV-8b, and CHN-SX-FAdV-4-1, were obtained from an outbreak of IBH in 35-day-old commercial layer chickens with 65% mortality and cultured in LMH cells. The liver from the infected chickens was homogenized and purified by filtration through a 0.22 µm syringe filter, and the filtered supernatant was inoculated into chicken hepatoma (LMH) cells. Cytopathic effect was observed daily under an inverted microscope (28). The supernatant was collected for virus identification by PCR and sequence analysis and stored at −80°C prior to inoculation for subsequent passages.

### Indirect immunofluorescence assay

LMH cells cultured in 24-well plates were infected with FAdV strains, and the immunofluorescence assay was conducted according to the manufacturer’s instructions. Goat anti-chicken IgG/FITC (Beijing Biosynthesis Biotechnology Co. Ltd, Beijing, China) with 1:100 dilution was used. Finally, the stained cells were photographed under a fluorescent microscope.

## RESULTS

### Gross lesions and histological examination

**Fig 1:**
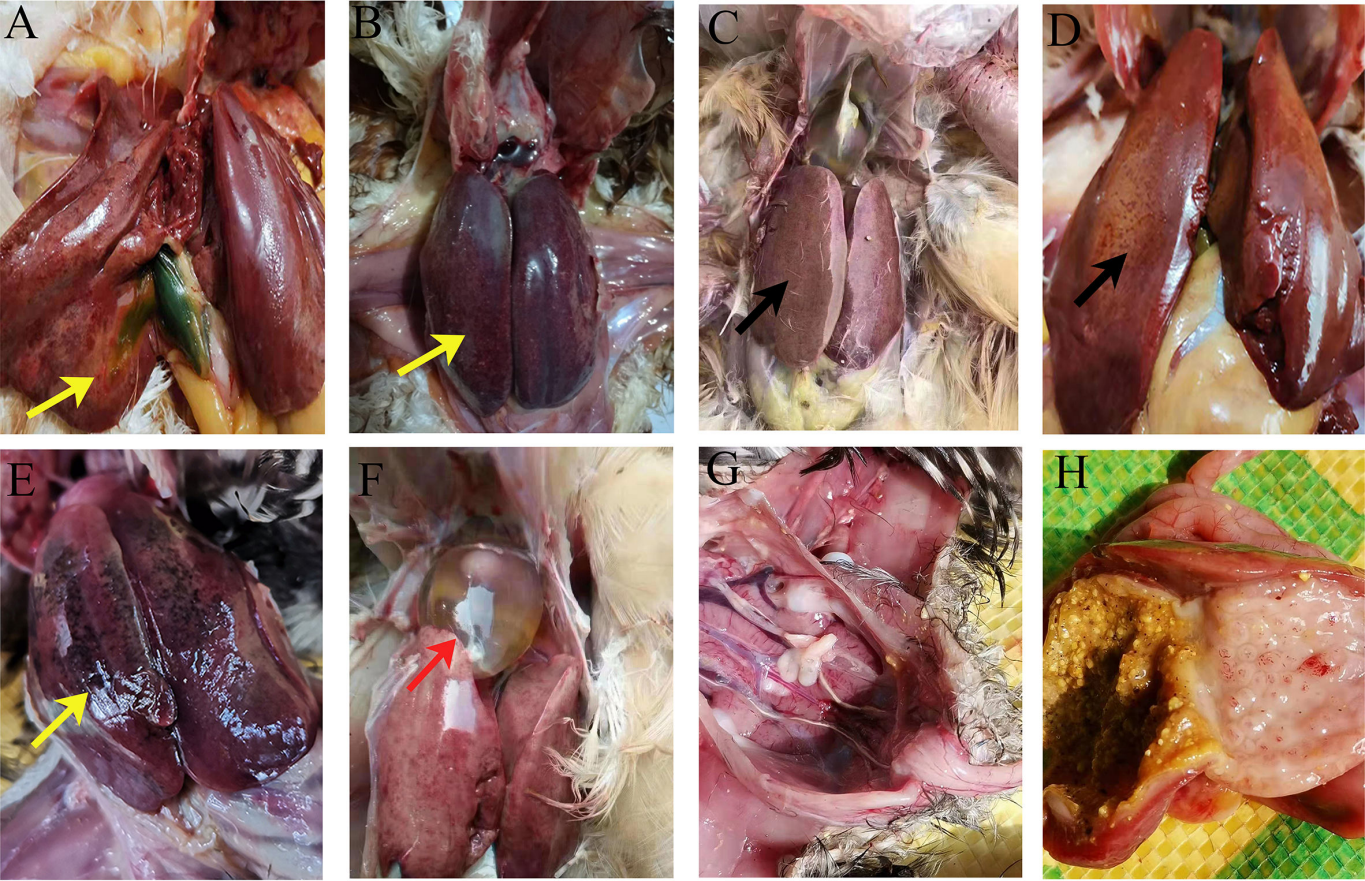
Gross pathological changes and histopathological analysis. (**A–F**) Representative images showing livers and fragile, necrotic, hemorrhagic (yellow arrow), enlarged liver with fatty degeneration (black arrow) and the heart with straw-colored fluid in the pericardial sac (red arrow). (**G**) The kidneys were congested and swollen with urate deposits. (**H**) Hemorrhagic spots on the papillae of the glandular stomach and diffuse hemorrhages at the junction of the glandular and muscular stomach.

**Fig 2:**
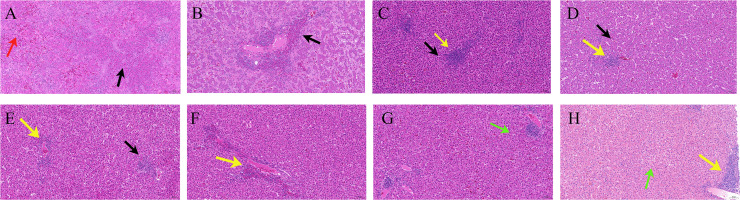
Histopathology of infected chicken liver tissue. (**A and B**) Disturbed hepatocyte arrangement with increased interstitial cells and focal infiltration of heterophilic granulocytes around a large number of vessels and in the liver parenchyma (black arrows); extensive hepatic sinusoidal bruising and dilatation (red arrows). (**C and D**) Extensive hepatocytes with mild cytoplasmic sparing (black arrows); more lymphocytes with a small number of heterophilic granulocytes (yellow arrows). (**E and F**) Hepatocytes with mild edema and loose, lightly stained cytoplasm (black arrows); heterophilic granulocytes and lymphocytes around the confluent area (yellow arrows). (**G and H**) Hepatocytes with mild steatosis and small round vacuoles in the cytoplasm (green arrows) (hematoxylin and eosin stain, scale bar = 50 µm).

Chickens from the affected house exhibited severely enlarged livers and fragile, hemorrhagic, and numerous yellow necrotic liver lesions (Fig. 1A through E). The individual chicken showed pericardial effusion, with about 10–20 mL of fluid in the pericardial sac (Fig. 1F), hemorrhagic spots on the papillae of the glandular stomach, and diffuse hemorrhages at the junction of the glandular and muscular stomach (Fig. 1H). The kidneys were congested and swollen with urate deposits (Fig. 1G). None of the chickens showed an enlarged bursa of Fabricius. Microscopic examination of livers demonstrated the presence of hemorrhages and severe hepatic degeneration and necrosis (Fig. 2A and B), hepatocellular disorders, hepatocellular enlargement, hepatocellular disintegration and necrosis, and slight hepatocyte fatty degeneration (Fig. 2G and H). In addition, lymphocytes with focal hyperplasia within liver lobules and focal aggregation of lymphocytes around the central were also observed in livers (Fig. 2C through F).

### Positive rate of different viruses

The RNA of genotype 3 avian HEV was detected in eight flocks, while the DNA of FAdV-4 was detected in 7/8 flocks, including six single and one mixed with FAdV-8a and 8b. Additionally, FAdV-8a was detected in 2/8 flocks, including one single and one mixed with FAdV-8b and 4, and FAdV-8b was detected in 1/8 flocks, which were co-infected with FAdV-8a and 4 (Table 1). For the detection of anti-avian HEV antibodies in the chickens in these farms, the positive rates ranged from 40% to 100% (Table 3).

**TABLE 3:** Prevalence of antibodies to avian HEV in chickens of different farms in the Shaanxi province, China in 2023

	Farm 1	Farm 2	Farm 3	Farm 4	Farm 5	Farm 6	Farm 7	Farm 8
ELISA (total antibodies) positive/no. tested (%)	25/30 (83%)	30/30 (100%)	29/30 (96%)	12/30 (40%)	16/30 (53%)	18/30 (60%)	21/30 (70%)	20/30 (66%)
HEV RNA positive samples/total analyzed (%)	21/30 (70%)	29/30 (96%)	28/30 (93%)	13/30 (43%)	18/30 (60%)	23/30 (76%)	14/30 (46%)	16/30 (53%)

### Sequence comparisons and phylogenetic tree construction

**Fig 3:**
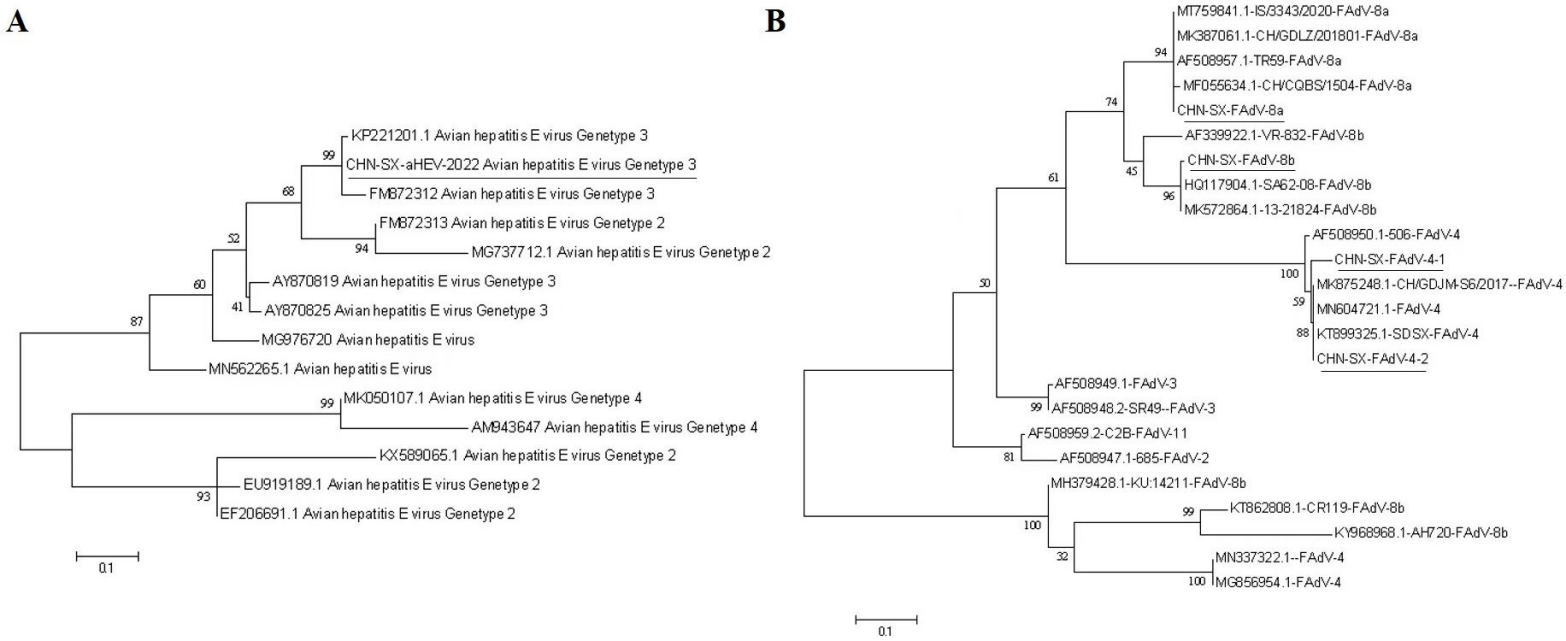
Phylogenetic trees constructed using the Neighbor-Joining method in MEGA7 software based on nucleotide sequence alignments. (**A**) Tree based on partial ORF2 gene of avian HEV (242 bp). (**B**) Tree based on the env gene of FAdV (896 bp). The number of nucleotide substitutions per 111 residues is given; the number of bootstrap trials is set to 1,000. The bar indicates genetic distance.

Four different partial FAdV hexon genes (896 bp) were obtained from all positive samples and designated as CHN-SX-XA001 to CHN-SX-XA004. Compared with corresponding hexon reference sequences, these sequences showed 98%–100%, 95%–99.8%, and 93%–100% identity with FAdV-8a, FAdV-8b and FAdV-4, respectively (Table 4). The sequences of CHN-SX-FAdV-8a, CHN-SX-FAdV-8b, CHN-SX-FAdV-4-1, and CHN-SX-FAdV-4-2 have been submitted to the GenBank, with the accession numbers OR824927, OR824928, OR824930, and OR824929. Phylogenetic analysis revealed that these sequences were closely related to FAdVs (Fig. 3B).

**TABLE 4:** Percent identities (%) between the FAdV isolates in the study and the known different genotypes FAdV strains in the GenBank based on 896 bases of hexon gene fragments

Isolates	Different genotypes of FAdV (% identity)		
	Genotype 8a	Genotype 8b	Genotype 4
CHN-SX-XA001	98–100	0	0
CHN-SX-XA002	0	95–99.8	0
CHN-SX-XA003 to 004	0	0	93–100

One of the 242 bp partial avian HEV ORF2 sequences was obtained from all positive samples, designated as CHN-SX-aHEV-1, and shared 74% to 97% identity with reference strains (Table 5). The highest identity (95%–97%) was found with genotype 3 isolates from the United States, Europe, and China (Table 5). The sequence of CHN-SX-aHEV-1 was submitted to GenBank (OR737846) and used to construct a phylogenetic tree with other known avian HEV strains obtained from GenBank. The results indicated that CHN-SX-aHEV-1 clustered with genotype 3 avian HEV (Fig. 3A).

**TABLE 5:** Percent identities (%) between the avian HEV isolate in the study and the known different genotypes avian HEV strains in the GenBank based on 242 bases of ORF2 fragments

Isolates	Different genotypes of avian HEV (% identity)			
	Genotype 1	Genotype 2	Genotype 3	Genotype 4
CHN-SX-aHEV-1	79–85	74–86	95–97	83–87

### Isolation and identification of the FAdV-8a/8b/4

**Fig 4:**
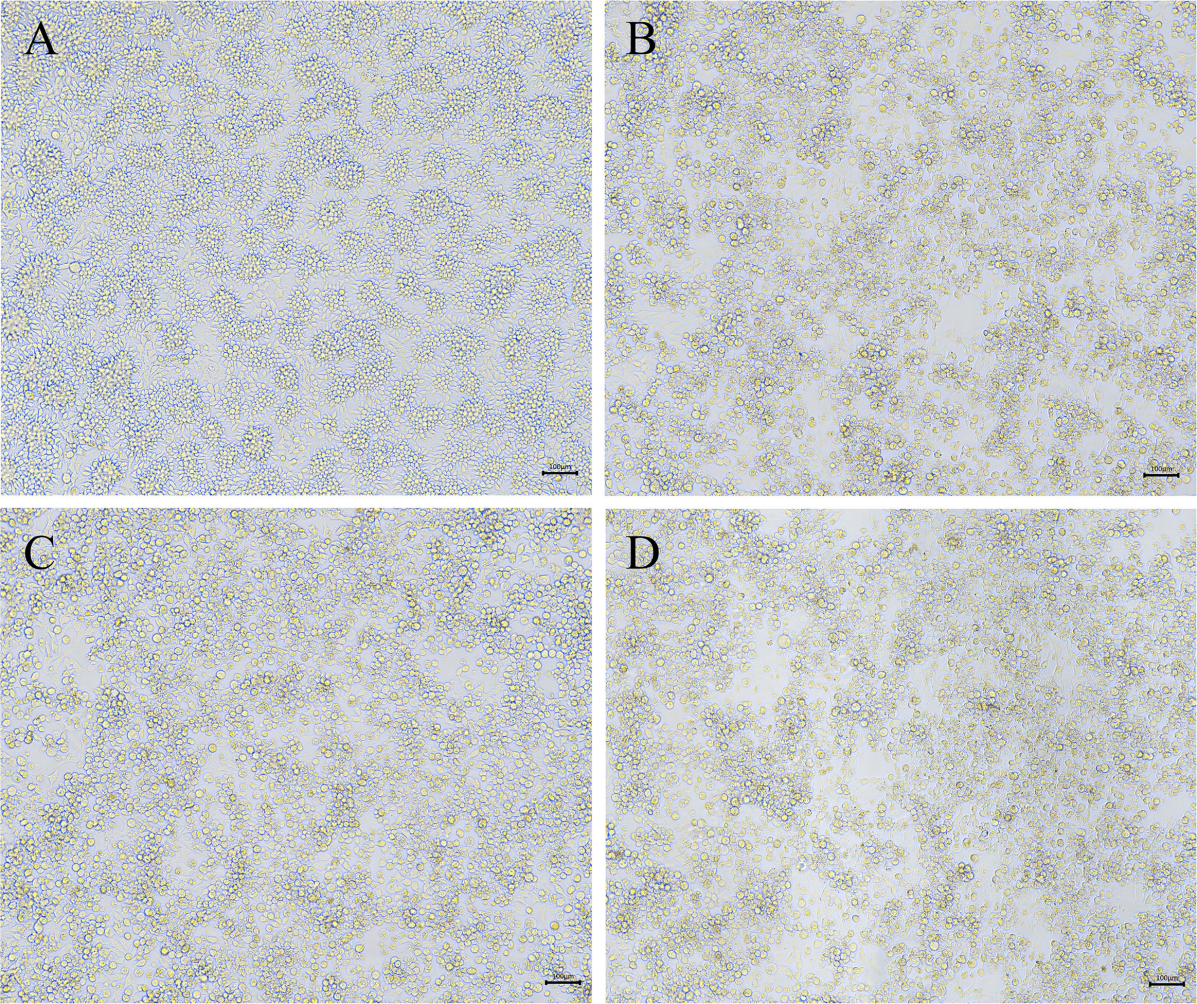
Cytopathological effects of LMH cells infected with FAdV-8a/8b/4 strain. Uninfected LMH cells (**A**). Infected cells are round and swollen (**B–D**). Scale bar, 100 µm.

**Fig 5:**
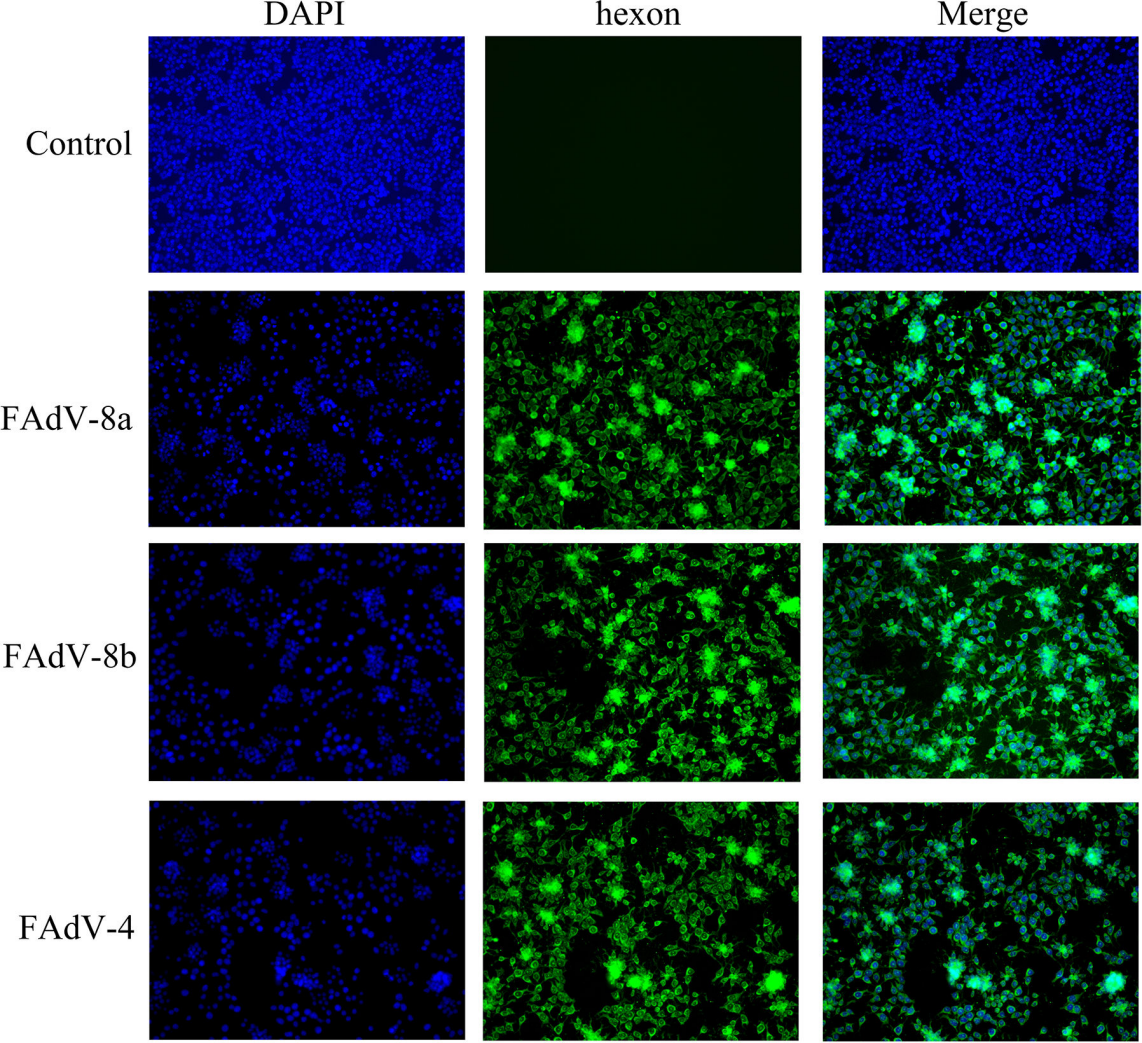
Fluorescence staining of LMH cells infected with FAdV. Non-infected LMH cells (first column). IFA result of LMH cells infected with FAdV-8a strain (second column), FAdV-8b strain (third column), and FAdV-4 strain (fourth column), respectively (200×).

FAdVs were isolated and serially passaged five times in LMH cells. Compared with negative control cells (Fig. 4A), the infected cells showed severe cytopathic effects, which consisted of enlarger and rounder of cells, the enhancement of intercellular space, and cell detachment (Fig. 4B through D). Meanwhile, the result of immunofluorescence assay (IFA) showed that a large number of immunofluorescent cells could be detected after IFA staining using FAdV-8a/8b/4 positive serum (Fig. 5). After five passages, FAdV hexon gene (896 bp) could be detected from collected LMH cells by PCR, and the sequences shared 100% identity with the original virus inoculum.

## DISCUSSION

Since 2015, FAdV infection has been endemic in poultry farms in China, particularly affecting broilers and layers aged 3–5 weeks. It was characterized by HHS (19, 29–31), leading to sudden death in a large number of chickens and significant economic losses for poultry farmers (32). Recently, FAdV infections have been described as coinfections involving pathogens such as ALV-J, IBDV, MDV, and avian HEV, and dual or multiple infections resulted in more severe clinical signs than FAdV infection alone (12, 19). In 2023, avian HEV and FAdV-8b coinfection caused increased deaths with a mortality rate of up to 40%, and liver enlargement rupture and splenomegaly in a Hy-line Brown parental breeder in Hebei Province, China (17). It has also been found that mixed infections with two or more FAdV serotypes were common in IBH and hydropericardium syndrome (HPS) cases (30). In China, FAdV infections have been caused by various FAdV species, with at least three species (C, D, and E) detected. Furthermore, two species of FAdVs (C and D) co-existed in one flock, and both were detected in samples collected from Jilin, Hubei, and Liaoning provinces, indicating that mixed infections by different FAdV species may occur in the same chicken flocks (30). In this study, avian HEV and FAdV were both detected in the diseased chickens, resulting in increased mortality (10%–65%), decreased egg production (10%–40%), pericardial effusion, liver hemorrhage, and enlarged livers and spleens in broiler breeder and laying hens. Combined with previous reports, coinfection of avian HEV and FAdV was popular in the flocks in China.

Hexon gene, a vital antigenic/structural protein of FAdV, contains various antigenic determinants that can differentiate between genotypes, species, and subspecies and is often used to analyze the genetic evolutionary relationship of FAdV (33, 34). It should be noted that various members of the 12 FAdV serotypes reported currently can induce inclusion body hepatitis syndrome (4), but the pathogenicity of different serotypes of viral strains was not identical. To date, the most severe condition infected by FAdVs was HHS caused by serotype 4, which was the main etiological agent associated with HHS and usually leads to high mortality (4, 35–37). In this study, the coinfection of FAdV and aHEV was identified on all eight farms, and the genotype of FAdV was not the same on each farm. Furthermore, it was found that coinfection, particularly dual infection, was common in farms with HHS, which demonstrated that mixed infections with a variety of viruses may be one of the major causes of FAdV outbreaks in chicken flocks in China. It is worth noting that one of the Hy-line layer flock mixed infection with avian HEV and FAdV-8a/b and 4 was described as suffering from increased mortality rate (68%), serious liver hemorrhage and necrosis, hepatomegaly, and gizzard erosions. Phylogenetic analysis of the FAdV hexon gene revealed that the clinically prevalent FAdV remained serotype 4 (FAdV-4), whereas other serotypes (FAdV-8a and 8b) appeared occasionally. Consequently, it is imperative that further research into the prevention and control strategies for FAdV-4 be undertaken with renewed emphasis.

Both avian HEV and FAdV can cause subclinical and persistent infections in chickens, resulting in important economic losses for the poultry industry (17). Currently, there are no effective vaccines or drugs for the prevention and treatment of these diseases. Since 2013, IBH-HPS induced by FAdV-4 has appeared in many provinces of China (10). To control the FAdV-4 epidemic, most flocks were inoculated with inactivated FAdV-4 vaccines and had some effects. However, the positive rate of FAdV in eight farms was still 65%, and the major genotype was FAdV-4, but other genotypes have also been found in this study. There are two main possible explanations for this finding. Firstly, there are numerous circulating strains of FAdV-4 in the field, and the current vaccines do not provide good protection. Secondly, other serotypes, such as FAdV, are also prevalent in the flocks, and cross-protection efficiency has limitations. Therefore, it is crucial to accelerate vaccine research for both diseases to establish the foundation for their prevention and control.

In summary, the disease may be mainly caused by FAdV and aHEV coinfection on these farms, and this is the first study to discover FAdV-4/8a/8b and avian HEV coinfection in a farm. The findings of this study provide valuable insights into the prevention and control of FAdV and avian HEV coinfection in chicken flocks.
